# Large Gliadin Peptides Detected in the Pancreas of NOD and Healthy Mice following Oral Administration

**DOI:** 10.1155/2016/2424306

**Published:** 2016-10-04

**Authors:** Susanne W. Bruun, Knud Josefsen, Julia T. Tanassi, Aleš Marek, Martin H. F. Pedersen, Ulrik Sidenius, Martin Haupt-Jorgensen, Julie C. Antvorskov, Jesper Larsen, Niels H. Heegaard, Karsten Buschard

**Affiliations:** ^1^The Bartholin Institute, Rigshospitalet, Copenhagen N, Denmark; ^2^Clinical Biochemistry, Immunology & Genetics, Statens Serum Institut, Copenhagen S, Denmark; ^3^The Hevesy Laboratory, DTU Nutech, Technical University of Denmark, Roskilde, Denmark; ^4^Institute of Organic Chemistry and Biochemistry, Academy of Sciences of the Czech Republic, Prague 6, Czech Republic; ^5^Enzyme Purification and Characterization, Novozymes A/S, Bagsværd, Denmark

## Abstract

Gluten promotes type 1 diabetes in nonobese diabetic (NOD) mice and likely also in humans. In NOD mice and in non-diabetes-prone mice, it induces inflammation in the pancreatic lymph nodes, suggesting that gluten can initiate inflammation locally. Further, gliadin fragments stimulate insulin secretion from beta cells directly. We hypothesized that gluten fragments may cross the intestinal barrier to be distributed to organs other than the gut. If present in pancreas, gliadin could interact directly with the immune system and the beta cells to initiate diabetes development. We orally and intravenously administered 33-mer and 19-mer gliadin peptide to NOD, BALB/c, and C57BL/6 mice and found that the peptides readily crossed the intestinal barrier in all strains. Several degradation products were found in the pancreas by mass spectroscopy. Notably, the exocrine pancreas incorporated large amounts of radioactive label shortly after administration of the peptides. The study demonstrates that, even in normal animals, large gliadin fragments can reach the pancreas. If applicable to humans, the increased gut permeability in prediabetes and type 1 diabetes patients could expose beta cells directly to gliadin fragments. Here they could initiate inflammation and induce beta cell stress and thus contribute to the development of type 1 diabetes.

## 1. Introduction

A gluten-free (GF) diet reduces the incidence of diabetes in nonobese diabetic (NOD) mice and DP-BB rats [1, 2]. In humans, early exposure to gluten-containing food has been associated with increased risk of islet autoimmunity [3], and a recent case study has described a prolonged remission period in a type 1 diabetes (T1D) patient adhering to the GF diet [4, 5]. Finally, up to 10% of T1D patients have coeliac disorders, compared to 1% of the background population, indicating a common pathogenesis in coeliac disease and T1D [6].

A gluten-containing diet affects immune cells in the pancreatic lymph nodes and possibly contributes to local inflammation. In healthy mice, gluten intake promotes a proinflammatory profile of regulatory T-cells in both mesenteric and pancreatic lymph nodes [7]. In BALB/c and NOD mice, we recently described changes in NK- and dendritic cell populations in pancreatic lymph nodes, when comparing GF- with a gluten-containing diet [8, 9]. However, whether the effects of gluten take place in the intestinal immune system or by direct priming in the local lymph nodes and pancreas is unknown.

Much evidences suggest that gliadin peptides cross the intestinal barrier. After gluten intake, large gliadin fragments are found in the small intestine due to partial resistance of gliadin to digestive enzymes [10, 11]. Intestinal permeability and serum zonulin levels are increased in T1D patients even before clinical onset of the disease [12, 13]. This may likely enhance the entry of gliadin fragments into lamina propria and lymphoid tissue. Finally, enterovirus infection, which is associated with T1D, increases the intestinal permeability [14]. After crossing the intestinal epithelium, it is likely that gliadin peptides enter the bloodstream. This is seen for other dietary proteins such as ovalbumin when administered orally to mice [15], and, in one study, gliadin has been demonstrated in serum and breast milk by ELISA [16], although the finding was never confirmed.

The current study investigates the murine uptake and biodistribution of 33-mer and 19-mer gliadin peptides. We used the proline-rich 33-mer (p56–88) and 19-mer (p31–49) alpha-gliadin peptides, which are resistant to digestive proteases [10, 11, 17] and widely studied due to their implication in coeliac disease (CD) [18, 19]. Their transepithelial passage in vitro is low in healthy individuals compared to CD patients, in whom the fragments are transported by protected transcellular transport [17, 20, 21]. We show that these large gliadin peptides are present in circulation after oral administration and that large gliadin fragments access pancreas even in nondiabetic BALB/c and C57BL/6 mice. This may contribute to local inflammation and beta cell stress, which could accelerate the development of type 1 diabetes.

## 2. Methods

### 2.1. Gliadin Peptides

The peptides H-LQLQPFPQPELPYPQPELPYPQPELPYPQPQPF-OHY (33-mer) and H-LGQQQPFPPQQPYPQPQPF-OHY (19-mer), 98% pure (Schafer-N, Denmark), were ^3^H-labeled in the underlined positions using diiodotyrosine (Y(3,5-I_2_)) iodinated peptides by standard technique [22, 23]. They were dissolved in DMSO, mixed with 10% palladium on carbon catalyst, and subjected to 10 Ci tritium gas in a tritium manifold system (RC Tritec) for 2 h at room temperature, then purified by HPLC, and conserved by addition of 50 mM ascorbic acid. pH was 7.5 for intravenous use (i.v.) and 6 for peroral (p.o.) use. Radiochemical stability was 10 days, during which the animal experiments were performed.

### 2.2. Mice

BALB/cA BomTac (males), C57BL/6JBomTac (males), and NOD/MrkTac mice were purchased from Taconic Europe A/S, Ejby, Denmark, kept in an SPF animal facility and fed standard Altromin 1324 diet. NOD mice were bred in the same facility. Animal experiments were approved by the Danish Animal Experiments Inspectorate and experiments performed according to international guidelines for the care and use of laboratory animals.

### 2.3. Liquid Chromatography-Mass Spectrometry (LC-MS)

BALB/c mice, 4–16 weeks of age, were given 650–900 *μ*g of 33-mer, either i.v. or p.o., and heparin-plasma was prepared 15–60 min later at 0°C. Protein inhibitors were added to the tissues that were homogenized, mixed with internal standard (GENESEQP:AZF58701), and precipitated with 50% methanol, 1% TFA, and centrifugation. The samples were analyzed using an Orbitrap XL (Thermo Scientific) equipped with a Nano LC (Easy nLC II, Thermo Scientific). The chromatographic system was a 10 cm, ID 75 *μ*m, 3 *μ*m C18-A2 column (Thermo Scientific) with a flow of 300 nL/min with 0.1% formic acid as mobile phase A and 0.1% formic acid in acetonitrile as B. The MS scan was performed using a resolution of 30000 and a scan range of 300–2000 *m*/*z*.

### 2.4. SDS-PAGE Analysis

Six mice received 230–1200 *μ*Ci of ^3^H-33-mer or ^3^H-19-mer i.v. or p.o. or 200 *μ*Ci of ^3^H-tyrosine (Perkin Elmer). Heparin-plasma was prepared from tail blood at 0°C and analysed by SDS-PAGE without further processing, after depletion of albumin and IgG using a commercial kit (Protea Biosciences) or after digestion with trypsin (incubation with 125 mM dithiothreitol, 0.05% SDS at 53°C, pH 7.5 for 45 min, followed by digestion with trypsin (Fluka) at 37°C). The gel was fixed in 15% formalin/25% ethanol and Coomassie-stained. For fluorography, the fixed gels were soaked in 7% glycerol and Amplify Fluorographic Reagent (GE Healthcare), each for 30 min, dried, and exposed on Amersham Hyperfilm MP at −80°C. Subsequently, the dried gels were rehydrated in 7% acetic acid for 2–4 h and Coomassie-stained.

For scintillation counting, gel slices were excised from the nonfixed gel, covered with 600 *μ*L of 30% hydrogen peroxide, and heated at 50°C overnight before measurement.

### 2.5. Matrix-Assisted Laser Desorption Ionization Time-of-Flight Mass Spectrometry (MALDI-TOF MS)

Plasma was fractionated in Amicon Ultra 10 kDa centrifugal filter units (Merck Millipore), Vivaspin 500, 5 kDa filter units (Sartorius), PD10 columns, Amicon Ultra 0.5 mL 10 kDa centrifugal filters, or SEP-PAK C18 Plus short cartridges (Waters), or they were ethanol-precipitated or acid-ethanol extracted. Slices from nonfixed gels were extracted overnight at 37°C in 100 *μ*L of 50 mM NH_4_CO_3_ with or without 12.5 ng/*μ*L Endoproteinase Glu-C Sequencing Grade (Roche Diagnostics). The gel slices were further extracted on a shaker for 15 min in 20 *μ*L extraction buffer (1 : 2 (vol/vol) 50 mM NH_4_HCO_3_/acetonitrile). The extracts were combined.

*S*amples were dried in a vacuum concentrator (Eppendorf 5301) and reconstituted in 20 *μ*L 5% acetic acid before desalting using POROS C18 matrix and elution onto a stainless steel 96-well MALDI target plate with 1 *μ*L *α*-cyano-4-hydroxycinnamic acid (HCCA matrix) ready-made from Agilent (6 mg/mL in 30% acetonitrile, 30% methanol, and 0.1% TFA) for dried droplet crystallization. Analysis was done on Bruker Ultraflex with Daltonics flexAnalysis software, externally calibrated using a standard peptide mixture (Bruker, range: 1,000–3,200 Da). Spectra were recorded in positive linear mode and summed from 100 laser shots.

The MS mass of 84 fragments >800 Da was calculated. They were randomly generated from the 33-mer sequence, allowing for deamidation of up to two glutamine residues (only one deamidation for Mr <1500 Da) and for sodium and potassium ion adduct formation. Measured masses were matched to the predicted masses, using a difference of <0.5 Da as threshold. Masses from negative control samples were matched using a threshold of 1.0 and removed.

### 2.6. Biodistribution

For initial evaluation of accumulation and elimination in blood, NOD mice (6–8 weeks) received 230–900 *μ*Ci ^3^H-33-mer or ^3^H-19-mer. For biodistribution experiments, BALB/c, C57BL/6, and NOD mice (8 or 12 weeks) received 50 *μ*Ci (1.5–3.6 nmol) of 33-mer or 19-mer, either p.o. or i.v., and NOD mice aged 20 weeks received 25 *μ*Ci (0.5 nmol) ^3^H-tyrosine. Organs were incubated with SOLVABLE (Perkin-Elmer) at 55°C until dissolved and then with 30% hydrogen peroxide to decolorize the solution. Ultima Gold (Perkin Elmer) was added, and samples were counted 1 h later in a Packard 1600TR scintillation counter.

Specific activities (dpm/mg tissue) were calculated by dividing the counts by the respective weight of the sample that was measured. The tissue distribution was examined using the tissue/blood ratios. To compare responses in blood across different doses (230–900 *μ*Ci), data were normalized to a dosage of 100 *μ*Ci.

For kinetics studies, using SDS-PAGE and fluorography, 230 *μ*Ci ^3^H-33-mer or 770 *μ*Ci of ^3^H-19-mer was used for i.v. administration and 620–1200 *μ*Ci ^3^H-33-mer was used for oral administration to NOD mice aged 6–8 weeks. One NOD mouse aged 20 weeks received 200 *μ*Ci ^3^H-tyrosine.

Before oral gavage, mice were starved for 4 h and were not fed until 20 min after.

### 2.7. Autoradiography

Standard tissue sections were dipped in a 1 : 1 dilution in water of melted Kodak NTB Emulsion with 10% Amplify Fluorographic Reagent (GE Healthcare), air-dried, stored in the dark at 4°C with desiccant for 1–3 weeks, developed in Kodak D19 developer, fixed in Ilford Rapid Fixer, HE stained, and examined in an Olympus Bx51 microscope (UPlanSApo 20x, 0.40) equipped with a Colorview 1 camera and Analysis getIT software (Olympus).

### 2.8. Statistical Analysis

One-way ANOVA with Tukey posttest was carried out using GraphPad Prism version 5.00 for Windows (GraphPad Software, San Diego, California, USA) to analyze the distribution of the label at each time point. The stomach and intestine were excluded from the analysis due to their large variation. The age of the animals (8 and 12 weeks) did not influence the uptake or distribution of radioactivity from the ^3^H-33-mer peptide. Thus, mice of different ages were pooled.

## 3. Results

**Figure 1:**
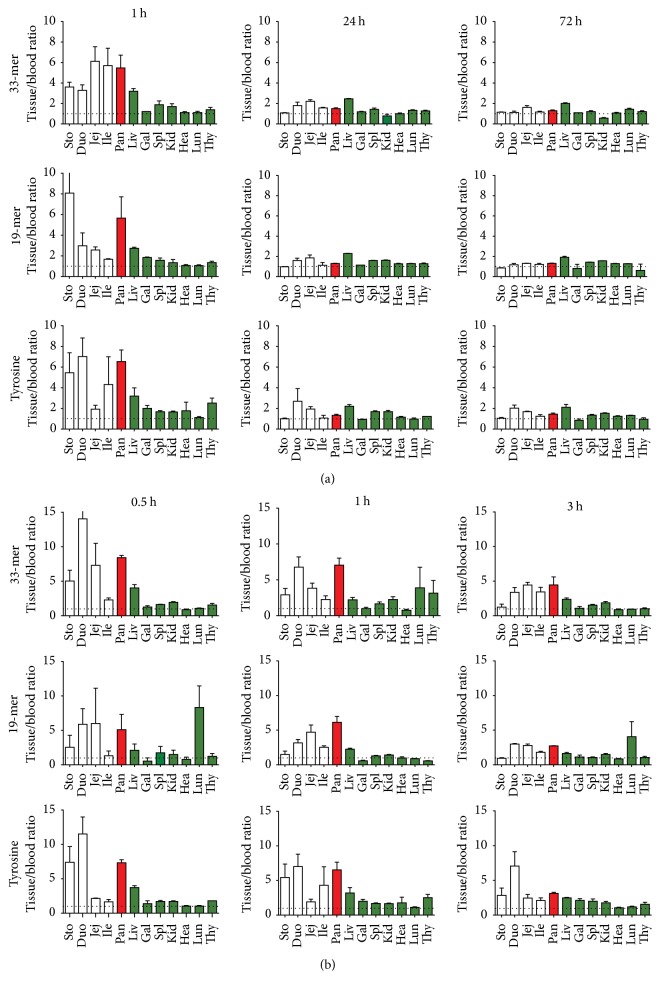
Quantification of ^3^H-33-mer, ^3^H-19-mer, and ^3^H-tyrosine following oral ingestion in NOD mice in intestine (white), pancreas (red), and other tissues (green). (a) Radioactivity relative to blood in selected tissues 1, 24, and 72 h after oral administration of ^3^H-33-mer to mice aged 8 and 12 weeks, of ^3^H-19-mer to mice aged 12 weeks, and of ^3^H tyrosine to mice aged 20 weeks (*N* = 2–4 mice). Error bars represent SEM. Sto (stomach), Duo (duodenum), Jej (jejunum), Ile (ileum), Pan (pancreas), Liv (liver), Gal (gallbladder), Spl (spleen), Kid (kidney), Hea (heart), Lun (lung), and Thy (thyroid gland). (b) Tissue distribution of ^3^H-33-mer, ^3^H-19-mer, and ^3^H-tyrosine tracer in NOD mice 0.5, 1, and 3 hours after oral administration. NOD mice (6–8 weeks) were given ^3^H-33-mer (upper), ^3^H-19-mer (middle), or ^3^H-labeled tyrosine (bottom). *N* = 4 mice. Error bars represent SEM. The abbreviations are as above.

**Figure 2:**
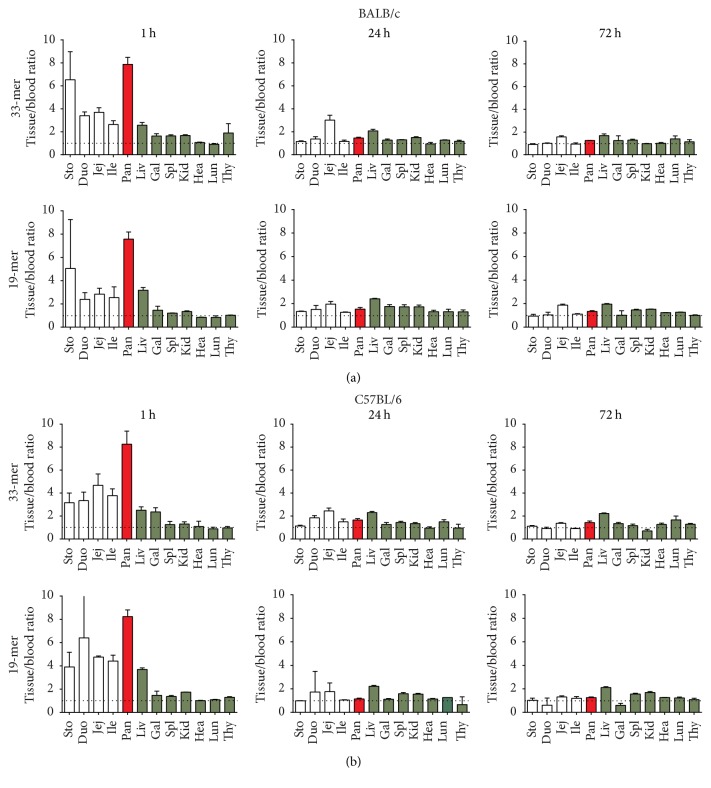
Tissue distribution of gliadin 33-mer and 19-mer following oral ingestion in BALB/c (a) and C57BL/6 mice (b), 8–12 weeks of age. Blood and organs were sampled after 1, 24, and 72 h. The specific radioactivity is shown relative to blood, and data are average values of 2–4 mice and shown with SEM values. Abbreviations are as in Figure 1.

To test the hypothesis that large gluten fragments might be absorbed from the intestine, two fragments that have been well investigated in coeliac disease, gliadin 33- and 19-mer, were tritium-labeled and given orally to NOD mice, and blood and tissues were sampled up to 72 hours after administration (Figure 1(a)). Interestingly, a high level of label from both fragments was seen in the pancreas shortly after administration (0.01 < *p* < 0.05). To increase the time resolution for this phenomenon, we looked in detail at the early events in NOD mice (Figure 2(b)) and found that the label was present 1/2 h after administration and still remained 3 hours after oral administration. A tyrosine control was included to simulate enzymatic degradation of the peptides, since the label was introduced into the peptides through this amino acid. This label accumulated similarly to the 33- and 19-mer, suggesting that degradation of the peptides could account for the accumulation of label.

To investigate if the intestinal transfer of tracer was specific for NOD mice, we also investigated BALB/c and C57BL/6 mice. In these strains, similar findings were seen (Figure 2), suggesting that the enteric permeability for gluten peptides was not specific for NOD mice and that the pancreatic accumulation of the label occurs similarly in several strains.

For reference, we investigated the absorbance and distribution of gliadin fragments following intravenous injection and observed an even more pronounced accumulation of label (Supp. Figure  1A (see Supplementary Material available online at http://dx.doi.org/10.1155/2016/2424306)). Again, the results did not differ when investigating the matter in BALB/c mice (Supp. Figure  1B).

**Figure 3:**
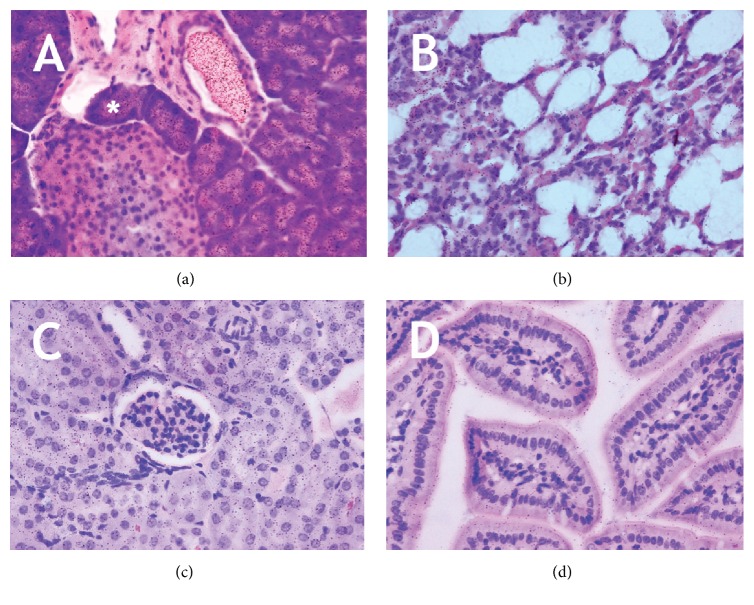
Autoradiography of mouse tissues following administration of ^3^H-33-mer. Mouse pancreas (a), lung (b), kidney (c), and ileum (d) were sampled 1 h after oral administration of ^3^H-33-mer. Asterisk denotes zymogen granules, which showed high accumulation of label. In all other tissues examined, homogenous labeling was observed. No difference was seen between i.v. and p.o. administration.

To investigate the spatial distribution of the radiolabel, we performed autoradiography. In pancreas (Figure 3(a)), the majority of the label was present in the zymogen granules of the exocrine pancreas and in the duct system and to a lesser extent in the islets. In the other tissues examined (lung (b), kidney (c), and ileum (d)), the label was less abundant and evenly distributed.

### 3.1. Analysis of Molecular Weight Species in the Blood

The results do not necessarily demonstrate the presence of the peptides in the various tissues, as the peptides might cleave during uptake [24]. This is in fact a distinct possibility, as investigated in Supp. Figure  2. In blood (Supp. Figure  2A), molecular weight species about twice the molecular weight of the peptide (12 kDa) were seen within minutes of injection. After 1 h, higher molecular weight species (HMW) emerged. Formation of the HMW species could be prevented by injecting unlabeled 33-mer, suggesting that the signals originated from 33-mer adsorbing to high molecular weight species or from tyrosine from the 33-mer that were incorporated into newly synthesized HMW molecules. The 19-mer (Supp. Figure  2B) showed similar behavior, except that the 12 kDa signals were absent at 30 min.

Blood analyses following oral administration showed similar results (Supp. Figure  2C), except again absence of the 12 kDa signals. It is noticeable that the 33-mer signal is still present in the blood, although in low amounts, 1 h after oral administration of the peptide. Interestingly, the 12 kDa signal, but not the HMW signal, also formed in vitro (Supp. Figure  2D). This suggests that the 12 kDa signal does not require de novo synthesis, whereas the HMW does.

To investigate the relation of the HMW signal with albumin and IgG, we selectively removed these molecules from blood samples from mice that had received 33-mer orally. This removed 35% of the label, regardless of the route of administration. If adding the ^3^H-33-mer to blood in vitro or if cold peptide was injected prior to injection of the labeled peptide, only 14-15% could be removed (Supp. Figure  2E). This suggests that the label was either associated with or synthesized into these molecules. Following trypsin digestion of serum, which degrades albumin, but not 33-mer (Supp. Figure  2F), no distinct band was released, suggesting that the HMW signals originate from de novo synthesis. Further, injection of ^3^H-tyrosine in mice results in appearance of the HMW band (data not shown). This is compatible with the delay in the appearance of the signal following oral ingestion (Supp. Figure  3A). In summary, our data are compatible with degradation of the 33-mer in vivo.

### 3.2. Mass Spectrometry Analysis of Blood and Tissue

**Figure 4:**
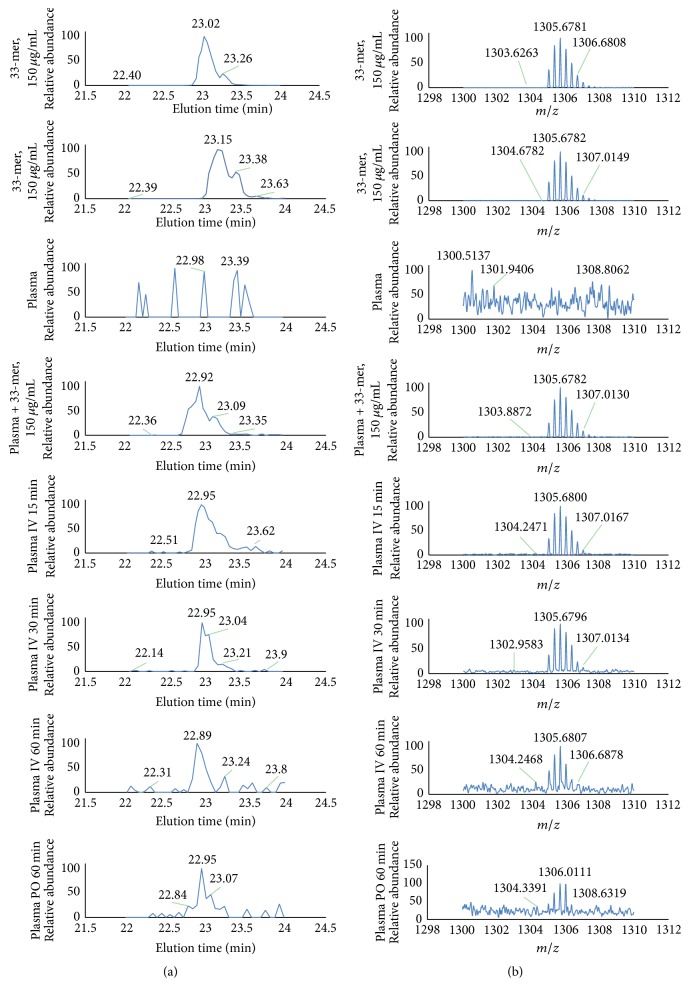
Analysis of plasma after administration of 33-mer to BALB/c mice by LC-MS. (a) Total ion current chromatograms with the 33-mer eluting at approximately 22.9 min. (b) Averaged mass spectra corresponding to the elution profiles. Plasma was precipitated with methanol-TFA before analysis. Rows 1–4 show control samples: 33-mer at 150 *µ*g/mL (rows 1 and 2), plasma (row 3), and plasma added 150 *µ*g/mL 33-mer (row 4). Rows 5–7 show plasma obtained 15, 30, and 60 min, respectively, after i.v. administration of 850 *µ*g 33-mer, and row 8 shows plasma obtained 60 min after p.o. administration of 650 *µ*g 33-mer. The levels after p.o. administration were close to the detection limit, and the peptide was not detected in all mice.

We finally used mass spectrometry to identify the peptides. We first optimized the LC-MS detection of the 33-mer by different purification procedures, using samples containing 33-mer, plasma, and plasma spiked with 33-mer (Figure 4). We then analyzed blood from BALB/c mice that had received 33-mer by intravenous or peroral administration and detected intact peptide in samples 15, 30, and 60 minutes after injection and 60 min after oral administration (Figure 4).

A total of 21 MALDI-TOF MS signals from blood and tissues could be matched to 33-mer fragments (Table 1). In blood, the longest peptide detected was a 32-mer, which was originally described as stable [11]. Using pancreas samples, the longest peptide observed was 16 amino acids. In NOD mice, we detected fragments in mice at 6, 8, 10, and 20 weeks of age. Further, we demonstrated that many of the fragments are also generated in vitro (Table 2) if 33-mer is incubated with mouse plasma, suggesting that the fragments form as a result of proteolytic enzymes found in plasma.

## 4. Discussion

In this report, we demonstrated that 33-mer and 19-mer gliadin peptides are readily absorbed from the intestine of NOD mice and healthy BALB/c and C57 mice. We did not find differences related to the age or strain of the animals.

The absorption of gliadin peptides is complex. Gliadin stimulates zonulin expression, which in turn enhances the uptake of 33-mer by the paracellular route [25], and absorption is also affected by cytokines and gut bacteria [26–28]. It could be expected that the absorption is increased during development of diabetes, as increased capillary permeability is seen in the gut [12, 13, 29, 30] and in the islets [31, 32] in type 1 diabetes patients, prediabetes patients, and relatives and in the BB rat model [33]. In NOD mice, the issue is less clear. Infection with a bacterial pathogen (*Citrobacter rodentium*) that can increase the intestinal permeability in NOD mice also increases disease incidence [34], but a change in permeability alone does not affect the disease mechanisms [35].

Nondegraded gliadin was previously identified in human breast milk [16] and in blood plasma in Wistar rats [26]. In addition, gliadin fragments have been demonstrated in animals under pathological conditions: in* Rhesus macaques* suffering from gluten-induced enteropathy or when sensitized to gluten [36]. Absorption of other macromolecules (ovalbumin and *β*-lactoglobulin) has been described, but in general the bioavailability is low and the half-life short of orally delivered peptide drugs and bioactive peptides [37, 38]. Therefore, our observation of 33-mer crossing the gut barrier in healthy mice is interesting in the light of the few similar reports describing dietary protein fragments of significant length, crossing the intestinal epithelium [39].

It is difficult to quantify the daily intake of 33-mer as the gliadin content in crop is variable, but based on data from [40] and, assuming that a mouse ingests 5 g of food per day, we estimate that the intake is 50–100 *μ*g. The native, amidated form of the 33-mer was not included in our studies, but since tissue transglutaminase is present in the intestine, it is likely that gliadin is at least partly deamidated before absorption. We therefore believe that the chosen dosages and peptides reflect the physiological exposure to gliadin.

We observed 10–20 kDa bands on SDS gels, which contained 33-mer and fragments hereof. The bands could be SDS-resistant, noncovalent di-, tri-, and multimers [41] in agreement with the ability of the (native, nondeamidated) 33-mer to dimerize and assemble into supramolecular structures, such as colloidal nanospheres [42]. Polymerized gliadin fragments could possibly accelerate the development of T1D, as multivalent surface presentation of epitopes [43] and particulate material [44] can more efficiently elicit immune responses. Self-assembly of the 33-mer might also protect it from enzymatic degradation or influence the uptake mechanism in the gut.

Other mechanism may, however, also explain the supernumerary gel bands. Deamidation of glutamines increases the number of negatively charged residues, thus decreasing the migration rate [45]. Prolyl cis-trans isomerization of gliadin fragments [46] could result in more rigid conformations that move slower in the gel [47] and could form as the 33-mer [42] and other gliadin sequences [48] are able to form PPII helix structure, which might exist at the denaturing conditions. In blood, the two latter phenomena could be promoted by enzymes (e.g., transglutaminases and prolyl isomerases), but spontaneous reactions are also possible.

The demonstration of gliadin fragments in islets provides new insight into how gliadin might contribute to diabetes development. The observation that gliadin readily or as a result of increased zonulin secretion [49] penetrates the gut, even in healthy animals and irrespective of the genetic background, suggests that gluten contributes to the pathogenesis of the disease. Thus, beta cells are most likely also exposed to the gliadin fragments as their molecular weight (4 kDa) is lower than capillaries retain [50]. This would cause stimulation of the insulin secretion, under both resting and glucose-stimulated conditions [51], and lead to beta cell stress. Further, gliadin contributes to local inflammation as it induces a more proinflammatory cytokine profile among T-cells in the intestinal and extraintestinal lymphatic centers [7], reduces the number of regulatory T-cells in BALB/c mice [52], stimulates NKG2D expression [53] and NK cell activity [8] in BALB/C and NOD mice, increases expression of dendritic cell activation markers in NOD mice [9], and, at least in vitro, activates macrophages [54]. These changes could all contribute to beta cell destruction.

How this suggested sequence of events translates into the human disease is not known, but several pathogenic aspects are similar in human and murine disease. For instance, gliadin also increases permeability in human intestinal mucosa [25], and recently a prolonged remission period among newly diagnosed T1D patients on a gluten-free, low-glycemic diet [55] was demonstrated. If the immunological consequences of gliadin exposure are similar in humans, the findings in the present study represent a missing link for understanding how gliadin contributes to the disease development.

## Supplementary Material

Fig Supp 1. Tissue distribution of 33-mer, 19-mer and tyrosine following intravenous injection in NOD and BALB/c mice, 8-12 weeks of age. 3H-labeled 33-mer or 3H-labeled 19-mer or 3H-tyrosine was administered to 20-week-old NOD mice. Blood and organs were sampled after 1, 24 and 72 h. The specific radioactivity is shown relative to blood, and data are average values of 2-4 mice and shown with SEM values. Abbreviations are as in Fig 1.Fig Supp 2. Kinetics of 3H-33-mer and 3H-19-mer in mouse blood following i.v. and p.o. administration. 3H-33-mer (A) or 3H-19-mer (B) was given i.v. to NOD mice (6-8 weeks). Tail blood was analyzed by SDS-PAGE and fluorography at the indicated time points (min). CT: control 33-mer/19-mer. In (A, right), 660 µg of unlabeled 33-mer was injected prior to the labeled peptide. (C) 3H-33-mer was administered p.o. to NOD mice (6-8 weeks). Blood was analyzed after 0.5, 1 and 20 h. All lanes were loaded with the same amount of radioactivity. CT is control 33-mer. (D) Fluorogram of in vitro incubation of blood plasma from a C57BL/6 mouse with 33-mer (28.6 ng/µl or 222.000 dpm/µl) for 0-4 h. CT is control 33-mer. (E) Plasma from mice, that had received 3H-labelled 33-mer one hour earlier by i.v. or p.o. administration, was depleted of albumin and IgG. Radioactivity counts in gel slices from lanes, loaded with plasma before (-) and after (+) extraction, are illustrated using pseudo-colors in intervals from <100 dpm (white), through 100-400 dpm, 400-1000 dpm and 1000-2000 dpm to >2000 dpm (dark gray). Data were normalized between lanes to contain identical amounts of radioactivity outside the albumin/IgG region. (F) Plasma from a mouse, that received 1.2 mCi 3H-labeled 33-mer p.o., was digested with trypsin followed by SDS-PAGE (right) and fluorography (left).

## Figures and Tables

**Table 1:** 33-mer fragments in mouse plasma and pancreas 45–60 min after oral administration, as determined by MALDI-TOF MS.

	LQLQPFPQPELPYPQPELPYPQPELPYPQPQPF	Sample
1	YPQPQPF	Plasma, NOD
2	FPQPELPY	Plasma, C57BL/6
3	QPFPQPELP	Plasma, NOD
4	ELPYPQPELP	Plasma, NOD pancreas, C57BL/6
5-1	LQLQPFPQPE	Pancreas, C57BL/6
5-2	QLQPFPQPEL	
6-1	QPFPQPELPY	Pancreas C57BL/6
6-2	ELPYPQPQPF	Plasma, NOD
7	QLQPFPQPELP	Plasma, NOD
8	ELPYPQPELPYP	Plasma, NOD pancreas, NOD
9	QLQPFPQPELPY	Plasma, NOD, C57BL/6
10	ELPYPQPELPYPQ	Plasma, NOD
11	YPQPELPYPQPQPF	Plasma, NOD, C57BL/6
12	LPYPQPELPYPQPQPF	Pancreas, NOD, C57BL/6
13	LQLQPFPQPELPYPQPE	Plasma, NOD
14	ELPYPQPELPYPQPELPYPQPQ	Plasma, NOD plasma, NOD
15-1	QPFPQPELPYPQPELPYPQPELPYPQPQ	Plasma, NOD, C57BL/6
15-2	LQLQPFPQPELPYPQPELPYPQPELPYP	
16-1	QPFPQPELPYPQPELPYPQPELPYPQPQP	Plasma, NOD, C57BL/6
16-2	LQPFPQPELPYPQPELPYPQPELPYPQPQ	
17	LQLQPFPQPELPYPQPELPYPQPELPYPQPQP	Plasma, C57BL/6

Mice (6–20 weeks) were given 500–900 *μ*g of 33-mer p.o. and blood and pancreas were analyzed. Samples were filtrates from 10 kDa centrifugal filters, supernatant from ethanol precipitation, an acidic ethanol extract from pancreas, peptide fractions from a PD10 desalting column, fractions from SEP-PAK C18 columns, and slices from SDS-PAGE gels (extracted without Glu-C digestion). The 33-mer fragment sequences were identified by matching the observed masses to the masses predicted for a list of arbitrary 33-mer fragments. Predictions were done using the theoretical masses and mass changes allowing 1-2 deamidations of glutamine and binding of sodium and potassium ions.

**Table 2:** Fragments formed in vitro during incubation of 33-mer in mouse plasma, as determined by MALDI-TOF MS.

	LQLQPFPQPELPYPQPELPYPQPELPYPQPQPF	Sample
1	YPQPQPF	Filtrate
2	ELPYPQPEL	Filtrate
3	QPFPQPELPY	Filtrate
4	YPQPELPYPQPQPF	Filtrate
5	ELPYPQPELPYPQPEL	Filtrate
6	LQLQPFPQPELPYPQPE	4–6 kDa
7	ELPYPQPELPYPQPELPY	Filtrate
8	ELPYPQPELPYPQPELPYPQPQPF	14–18 kDa
9	FPQPELPYPQPELPYPQPELPYPQPQPF	10–12 kDa
10	PFPQPELPYPQPELPYPQPELPYPQPQPF	10–12 kDa
11	LQLQPFPQPELPYPQPELPYPQPELPYPQP	14–18 kDa
12	QPFPQPELPYPQPELPYPQPELPYPQPQPF	14–18 kDa
13	QLQPFPQPELPYPQPELPYPQPELPYPQPQP	14–17 kDa
14	LQLQPFPQPELPYPQPELPYPQPELPYPQPQ	14–17 kDa
15	LQLQPFPQPELPYPQPELPYPQPELPYPQPQP	4–6 kDa
16	LQLQPFPQPELPYPQPELPYPQPELPYPQPQPF	4–6 kDa, 6–9 kDa, 14–17 kDa

33-mer was incubated at 37°C in mouse plasma, and samples were filtered through 5 kDa MWCO centrifugal filters or run on SDS-PAGE gels before MALDI-TOF MS analysis. Approximate ranges of the gel slices, according to the molecular weight marker, are shown, and assignment of observed masses to 33-mer fragments was done as described in Table 1. The appearance of bands at 10–20 kDa on SDS-PAGE gels during incubation was confirmed before analysis of gel slices (not shown). The identified fragments at 10–18 kDa were not found in plasma where 33-mer was not added or when sampled at *t* = 0.
