# Corrigendum to “Large Gliadin Peptides Detected in the Pancreas of NOD and Healthy Mice following Oral Administration”

**DOI:** 10.1155/2017/9709704

**Published:** 2017-03-12

**Authors:** Susanne W. Bruun, Knud Josefsen, Julia T. Tanassi, Aleš Marek, Martin H. F. Pedersen, Ulrik Sidenius, Martin Haupt-Jorgensen, Julie C. Antvorskov, Jesper Larsen, Niels H. Heegaard, Karsten Buschard

**Affiliations:** ^1^The Bartholin Institute, Rigshospitalet, Copenhagen N, Denmark; ^2^Clinical Biochemistry, Immunology & Genetics, Statens Serum Institut, Copenhagen S, Denmark; ^3^The Hevesy Laboratory, DTU Nutech, Technical University of Denmark, Roskilde, Denmark; ^4^Institute of Organic Chemistry and Biochemistry, Academy of Sciences of the Czech Republic, Prague 6, Czech Republic; ^5^Enzyme Purification and Characterization, Novozymes A/S, Bagsværd, Denmark

In the article titled “Large Gliadin Peptides Detected in the Pancreas of NOD and Healthy Mice following Oral Administration” [1], there was an error in the peptide sequences in Section 2.1. Gliadin Peptides, which should be corrected as follows:

The sequences H-LQLQPFPQPELPYPQPELPYPQPELPYPQPQPF-OHY and H-LGQQQPFPPQQPYPQPQPF-OHY should be corrected to H-LQLQPFPQPELPYPQPELPYPQPELPYPQPQPF-OH and H-LGQQQPFPPQQPYPQPQPF-OH.
