# Supplementary figures and images for: Large Gliadin Peptides Detected in the Pancreas of NOD and Healthy Mice following Oral Administration
Source: J Diabetes Res. 2016 Oct 4;2016:2424306. doi: 10.1155/2016/2424306 (PMC5067331; doi:10.1155/2016/2424306)

SUPP FIG 1


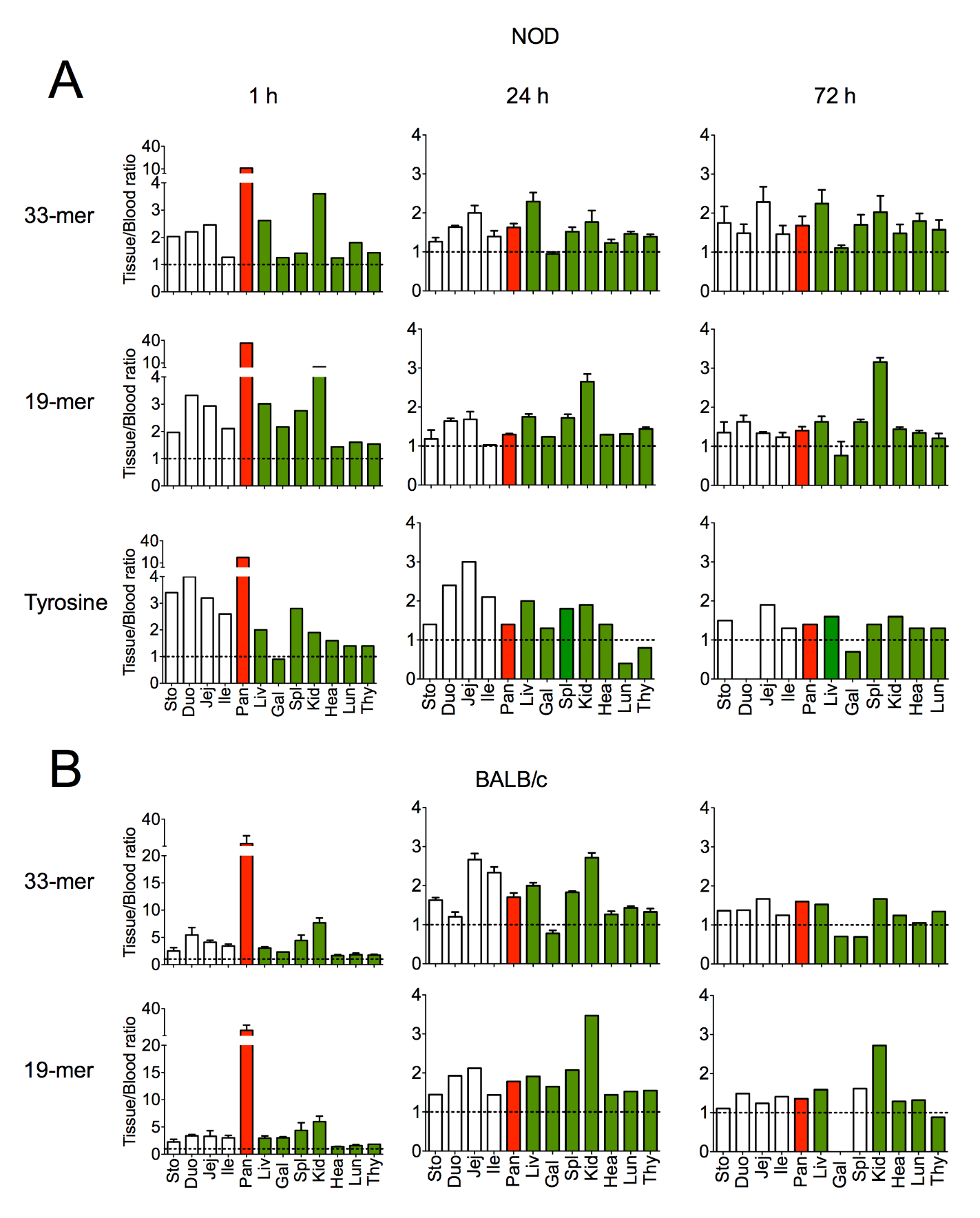


SUPP FIG 2


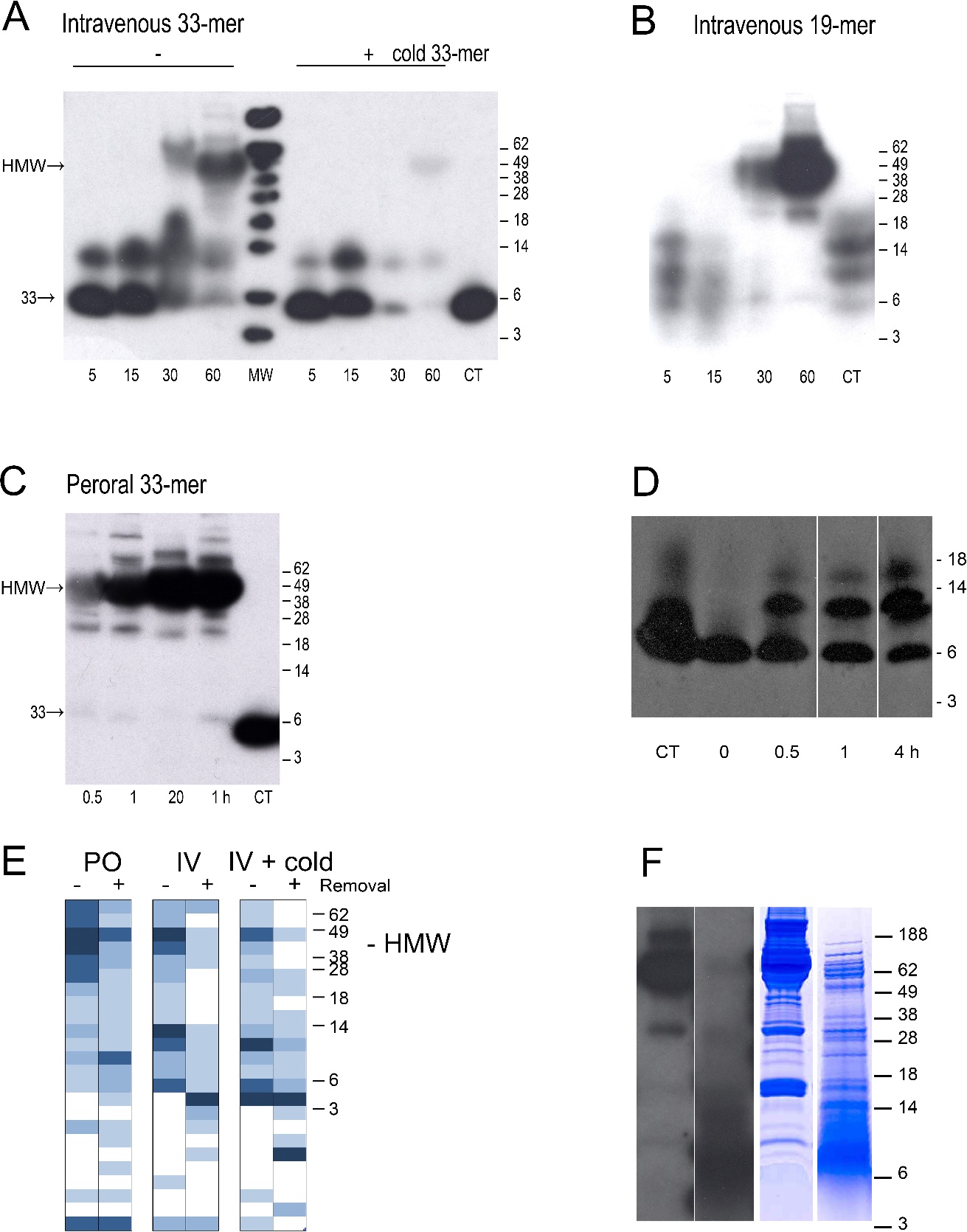

Supplement: Supplementary file 1 — Fig Supp 1. Tissue distribution of 33-mer, 19-mer and tyrosine following intravenous injection in NOD and BALB/c mice, 8-12 weeks of age. 3H-labeled 33-mer or 3H-labeled 19-mer or 3H-tyrosine was administered to 20-week-old NOD mice. Blood and organs were sampled after 1, 24 and 72 h. The specific radioactivity is shown relative to blood, and data are average values of 2-4 mice and shown with SEM values. Abbreviations are as in Fig 1.Fig Supp 2. Kinetics of 3H-33-mer and 3H-19-mer in mouse blood following i.v. and p.o. administration. 3H-33-mer (A) or 3H-19-mer (B) was given i.v. to NOD mice (6-8 weeks). Tail blood was analyzed by SDS-PAGE and fluorography at the indicated time points (min). CT: control 33-mer/19-mer. In (A, right), 660 µg of unlabeled 33-mer was injected prior to the labeled peptide. (C) 3H-33-mer was administered p.o. to NOD mice (6-8 weeks). Blood was analyzed after 0.5, 1 and 20 h. All lanes were loaded with the same amount of radioactivity. CT is control 33-mer. (D) Fluorogram of in vitro incubation of blood plasma from a C57BL/6 mouse with 33-mer (28.6 ng/µl or 222.000 dpm/µl) for 0-4 h. CT is control 33-mer. (E) Plasma from mice, that had received 3H-labelled 33-mer one hour earlier by i.v. or p.o. administration, was depleted of albumin and IgG. Radioactivity counts in gel slices from lanes, loaded with plasma before (-) and after (+) extraction, are illustrated using pseudo-colors in intervals from <100 dpm (white), through 100-400 dpm, 400-1000 dpm and 1000-2000 dpm to >2000 dpm (dark gray). Data were normalized between lanes to contain identical amounts of radioactivity outside the albumin/IgG region. (F) Plasma from a mouse, that received 1.2 mCi 3H-labeled 33-mer p.o., was digested with trypsin followed by SDS-PAGE (right) and fluorography (left). [file 2424306.f1.docx]
